# Supplementary material for: Resolving the full spectrum of human genome variation using Linked-Reads
Source: Genome Res. 2019 Apr;29(4):635–45. doi: 10.1101/gr.234443.118 (PMC6442396; doi:10.1101/gr.234443.118)
Supplement: Supplemental Material [file supp_gr.234443.118_Supplemental_longranger-2.2.zip › longranger-2.2.tar.bz2!/lr2.2-public/lib/stan/cmdstan/stan/doc/api/html/math_2functions_2lbeta_8hpp_source.html]

Stan: src/stan/math/functions/lbeta.hpp Source File


|  |  |
| --- | --- |
|  | Stan  2.5.0  probability, sampling & optimization |


- Main Page
- Related Pages
- Namespaces
- Classes
- Files

- File List
- File Members

All Classes Namespaces Files Functions Variables Typedefs Enumerations Enumerator Friends Macros Pages

- src
- stan
- math
- functions

lbeta.hpp

Go to the documentation of this file.

1 #ifndef STAN\_\_MATH\_\_FUNCTIONS\_\_LBETA\_HPP

2 #define STAN\_\_MATH\_\_FUNCTIONS\_\_LBETA\_HPP

3

4 #include <boost/math/tools/promotion.hpp>

5

6 #include <boost/math/special\_functions/gamma.hpp>

7

8 namespace stan {

9  namespace math {

10

57  template <typename T1, typename T2>

58  inline typename boost::math::tools::promote\_args<T1,T2>::type

59  lbeta(const T1 a, const T2 b) {

60  using boost::math::lgamma;

61  return lgamma(a)

62  + lgamma(b)

63  - lgamma(a + b);

64  }

65

66  }

67 }

68

69 #endif

stan::agrad::lgamma

fvar< T > lgamma(const fvar< T > &x)

**Definition:** lgamma.hpp:15

stan::math::lbeta

boost::math::tools::promote\_args< T1, T2 >::type lbeta(const T1 a, const T2 b)

Return the log of the beta function applied to the specified arguments.

**Definition:** lbeta.hpp:59

stan::math::lgamma

double lgamma(double x)

**Definition:** lgamma.hpp:31


---

[ Stan Home Page ]

*© 2011–2014,
Stan Development Team.*
